# Neuritogenic Monoglyceride Derived from the Constituent of a Marine Fish for Activating the PI3K/ERK/CREB Signalling Pathways in PC12 Cells

**DOI:** 10.3390/ijms141224200

**Published:** 2013-12-12

**Authors:** Wei Yang, Yan Luo, Ruiqi Tang, Hui Zhang, Ying Ye, Lan Xiang, Jianhua Qi

**Affiliations:** College of Pharmaceutical Sciences, Zhejiang University, Yu Hang Tang Road 866, Hangzhou 310058, China; E-Mails: 21119036@zju.edu.cn (W.Y.); 10919011@zju.edu.cn (Y.L.); ricky.tang@163.com (R.T.); 21319043@zju.edu.cn (H.Z.); 21119037@zju.edu.cn (Y.Y.)

**Keywords:** 1-*O*-(myristoyl) glycerol, neuritogenic activity, *Ilisha elongate*, PC12 cells, 1-*O*-(stearoyl) glycerol, ERK

## Abstract

A neuritogenic monoglyceride, 1-*O*-(myristoyl) glycerol (MG), was isolated from the head of *Ilisha elongate* using a PC12 cell bioassay system, and its chemical structure was elucidated using spectroscopic methods. MG significantly induced 42% of the neurite outgrowth of PC12 cells at a concentration of 10 μM. To study the structure-activity relationships of MG, a series of monoglycerides was designed and synthesised. Bioassay results indicated that the alkyl chain length plays a key role in the neuritogenic activity of the monoglycerides. The groups that link the propane-1,2-diol and alkyl chain were also investigated. An ester linkage, rather than an amido one, was found to be optimal for neuritogenic activity. Therefore, 1-*O*-(stearoyl) glycerol (SG), which induces 57% of the neurite outgrowth of PC12 cells at 10 μM, was determined to be a lead compound for neuritogenic activity. We then investigated the mechanism of action of neurite outgrowth induced by SG on PC12 cells using protein specific inhibitors and Western blot analysis. The mitogen-activated kinase/ERK kinase (MEK) inhibitor U0126 and the phosphatidylinositol-3 kinase (PI3K) inhibitor LY294002 significantly decreased neurite outgrowth. At the same time, SG increased phosphorylation of CREB in protein level. Thus, SG-induced neuritogenic activity depends on the activation of the extracellular-regulated protein kinase (ERK), cAMP responsive element-binding protein (CREB) and PI3K signalling pathways in PC12 cells.

## 1. Introduction

With the development of aging society, Alzheimer’s disease (AD) has become a leading cause of dementia in the older population. The costs associated with treating dementia worldwide totaled over $600 billion in 2010 [1]. The most popular selling drugs currently approved by the Food and Drug Administration (USA) for AD treatment include donepezil, rivastigmine, galantamine and memantine. Donepezil, rivastigmine and galantamine are cholinesterase inhibitors, whilst memantine is an *N*-methyl-d-aspartic acid (NMDA) receptor antagonist [2]. However, these drugs can only improve symptoms or slow down AD progression, not cure the disease [3].

Neurotrophic factors are considered potential therapeutic drugs for the treatment of neurodegenerative disorders. The targeted delivery of nerve growth factor (NGF) to basal forebrain cholinergic neurons has been reported to prevent cell death, stimulate synaptic cholinergic function and promote cognitive improvement in animals [4,5]. However, the application of NGF as a drug is limited because it cannot cross the blood brain barrier (BBB). Thus, finding small molecules that have neurotrophic properties similar to those of NGF (NGF mimics) [6–8] and can cross the BBB is of great importance. Natural products are a great source of compounds for medical uses. Many potential lead compounds against AD have been found from natural products on land [9–12], however, marine organisms remain largely untapped for their potential for AD treatment [13]. Thus, isolation of active compounds from marine sources and determination of lead compounds for AD drug development are worthwhile endeavors.

In our laboratory, the PC12 cell bioassay system has long been established as a reliable bioassay system for the investigation of potential drugs with NGF mimicking activity. During our screening of active samples from marine natural products, the head of a fish, *Ilisha elongata*, was found to show neuritogenic activity against PC12 cells. *I. elongate*, belonging to family Pristigasteridae, is one of the main commercial fishes in coastal fisheries of the Indian Ocean, East China Sea and Russian waters [14–16]. In this paper, we report the isolation, structural elucidation and biological activity of MG obtained from the head of *I. elongate*. We further synthesised a series of compounds based on the structure of MG and obtained the lead compound SG by studying the structure-activity relationships (SARs) of the monoglycerides. The mechanism of action of neurite outgrowth induced by SG on PC12 cells was finally investigated.

## 2. Results and Discussion

### 2.1. Isolation and Structural Elucidation of 1-*O*-(Myristoyl) Glycerol

**Figure 1.**
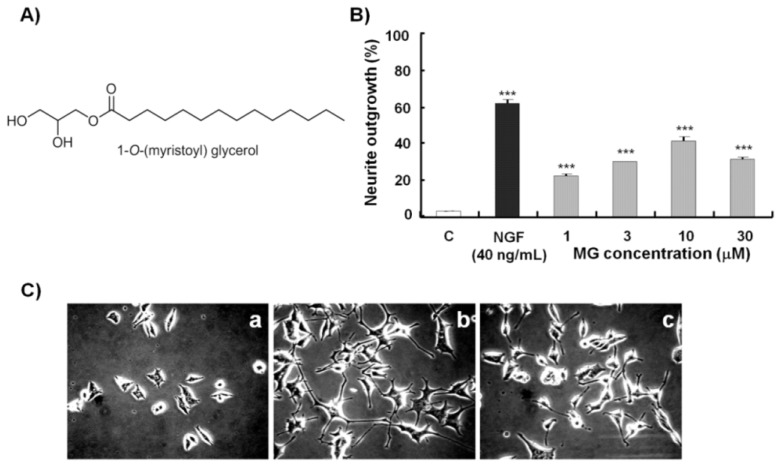
Chemical structure of MG, dose-dependent responses and photomicrographs of the NGF mimicking activity of MG 48 h after treatment. (**A**) Chemical structure of MG; (**B**) Percentage of neurite outgrowths of PC12 cells treated with MG at concentrations of 1, 3, 10 and 30 μM. C: solvent control (0.5% DMSO); NGF (40 ng/mL): positive control; and (**C**) Photomicrographs of PC12 cells obtained under a phase-contrast microscope 48 h after treatment: (**a**) solvent control (0.5% DMSO); (**b**) NGF (40 ng/mL); (**c**) MG (10 μM). Independent experiments were repeated three times. Each value represents the mean ± SEM of three replicates. ** and *** indicate significant differences relative to the control at *p* < 0.01 and *p* < 0.001, respectively.

The head of *I. elongate* was freeze-dried, ground to a powder and extracted with methanol to obtain a crude extract. The extract was partitioned between EtOAc and H_2_O. The active EtOAc layer was subjected to silica open-column chromatography. The active fraction contained monoglyceride derivatives, as determined by high-resolution (HR)-ESI-TOF LC/MS analysis. The derivative with the highest content of active monoglycerides was obtained by HPLC purification. Only this compound was adequate for structural elucidation. The compound was identified as MG by spectroscopic analysis, and its spectroscopic data were comparable with those reported in the literature (Figure 1A) [17].

### 2.2. NGF Mimic Activity of 1-*O*-(Myristoyl) Glycerol

The NGF mimicking activity of MG was tested on PC12 cells using NGF as a positive control and dimethyl sulfoxide (DMSO) as a negative control. Figure 1B shows the percentage of neurite outgrowth of PC12 cells induced by MG at concentrations ranging from 1 to 30 μM. MG showed dose-dependent increases in activity from 22% to 42% at concentrations ranging from 1 to 10 μM 48 h after treatment. At 30 μM, cytotoxicity was observed and NGF mimicking activity dropped to less than 30%. The solvent control induced a small number of neurite outgrowths. When treated with 10 μM MG, the cells showed long bipolar neurite outgrowths 48 h after treatment (Figure 1C).

### 2.3. Synthesis of 1-*O*-(myristoyl) Glycerol Derivatives

MG was isolated from the head of *I. elongata*. HR-ESI-TOF LC/MS analysis showed several other peaks near the MG peak, which suggests the presence of MG derivatives. However, the weight of these derivatives was inadequate for structural elucidation. Given that isolation of many compounds from natural sources is far more difficult than that obtained from synthetic means, we obtained MG derivatives for SAR studies through chemical synthesis.

**Figure 2.**
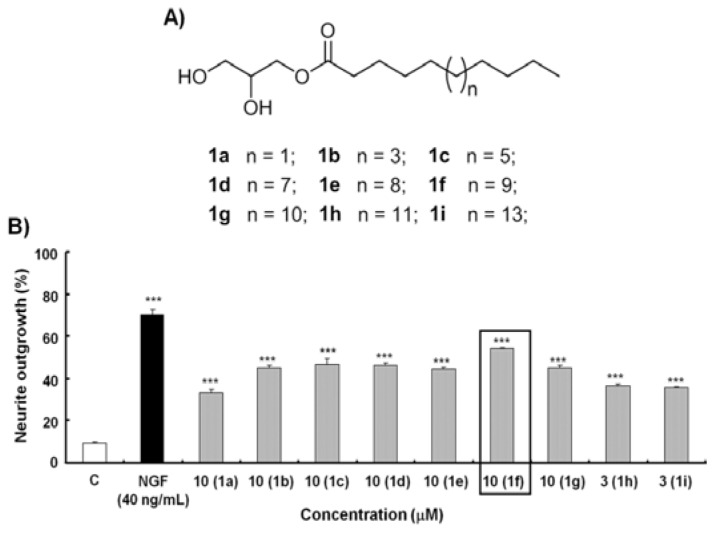
Chemical structures of the derivatives and their NGF mimicing activity. (**A**) Chemical structures of **1a**–**1i**; and (**B**) Percentage of neurite outgrowths of PC12 cells induced by compounds **1a**–**1i** at the optimum concentration 48 h after treatment. “*n*” indicates the number of the carbon atoms and *** represents significant differences relative to the control at *p* < 0.001.

First, the structural design of the monoglycerides focused on the length of the alkyl chain to determine the optimum alkyl chain length (Figure 2A). Monoglyceride derivatives **1a**–**1d** (C_10_, C_12_, C_14_, C_16_), **1f** (C_18_), **1h** (C_20_) and **1i** (C_22_) with different alkyl chain lengths were synthesized. Biological activity results showed that **1f** has the best activity (54%) at 10 μM. To determine the exact alkyl chain length of the monoglycerides, compounds **1e** (C_17_) and **1g** (C_19_), with carbon atom numbers of 17 and 19, respectively, were synthesized. Neuritogenic activity results showed that **1f** has slightly higher activity than either **1e** or **1g** at 10 μM (Figure 2B). These findings suggest that the alkyl chain length affects the NGF mimicking activity of the monoglycerides. The monoglyceride with an alkyl chain length of 18 carbon atoms (**1f**) was thus determined to be the optimal structure for neuritogenic activity.

**Figure 3.**
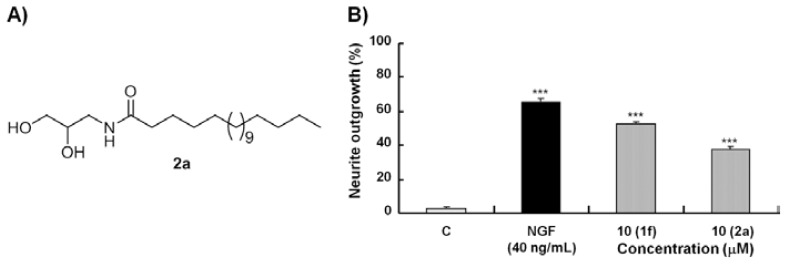
Chemical structure of **2a** and the neuritogenic activity of monoglyceride derivatives with different linkages. (**A**) Chemical structure of **2a**; and (**B**) Percentage of neurite outgrowths of PC12 cells induced by **1f** and **2a** at their optimal concentrations 48 h after treatment. *** indicates significant differences relative to the control at *p* < 0.001.

**Figure 4.**
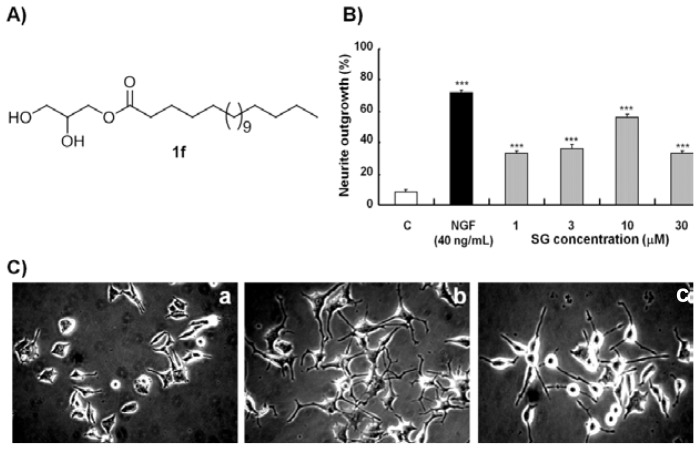
Chemical structure and NGF mimicking activity of SG. (**A**) Chemical structure of SG; (**B**) Percentage of neurite outgrowths of PC12 cells treated with SG at concentrations of 1, 3, 10 and 30 μM. C: solvent control (0.5% DMSO); NGF (40 ng/mL): positive control; and (**C**) Photomicrographs of PC12 cells obtained under a phase-contrast microscope: (**a**) solvent control (0.5% DMSO); (**b**) NGF (40 ng/mL); (**c**) **1f** (10 μM). *** indicates significant differences relative to the control at *p* < 0.001.

Aside from the alkyl chain length, the linkage group is also believed to play an important role in the neuritogenic activity based on our previous results [18]. After the determination of the optimal length of the alkyl chain, the ester linkage group between the glycerol and alkyl chain of **1f** was replaced by an amide bond. Compound **2a**, with an amido linkage and 18 carbon atoms on the alkyl chain, was synthesized (Figure 3A). The percentages of neurite outgrowths induced by **1f** and **2a** were 52% and 37%, respectively, at the optimal concentration (Figure 3B). SG (**1f**) with 18 carbon atoms on the alkyl chain and an ester linkage showed the best neuritogenic activity toward PC12 cells amongst all of the synthesized compounds. Thus, SG (**1f**) was determined as a lead compound (Figure 4A).

The dose-dependent activity of SG was investigated at concentrations ranging from 1 to 30 μM (Figure 4B). At 10 μM, SG showed a maximum NGF mimicking activity of 57%. Even at 1 μM, SG significantly induced neurite outgrowth (*p* < 0.001). Figure 4C shows morphological changes in PC12 cells treated with SG at 10 μM after 48 h.

### 2.4. Mechanism of Action of 1-*O*-(Stearoyl) Glycerol

The mechanism of action of NGF-induced neuritogenesis has been well established [19–21]. NGF binds to the tropomyosin-related kinase A, which is a transmembrane specific receptor for inducing specific tyrosine residues located at the intracellular domain phosphorylate and activating a number of kinases. Amongst them, the PI3K/Akt and the mitogen-activated protein kinase/ERK signalling pathways are known to significantly affect NGF-induced neurite outgrowth and neuroprotection [22,23]. To understand the NGF mimicking activity of SG and evaluate the cytotoxicity of the compound at the cellular level, we used inhibitors of specific proteins related to NGF signalling pathways and the 3-(4,5-dimethylthiazol-2-yl)-2,5-diphenyltetrazol-ium bromide (MTT) assay for the investigation.

**Figure 5.**
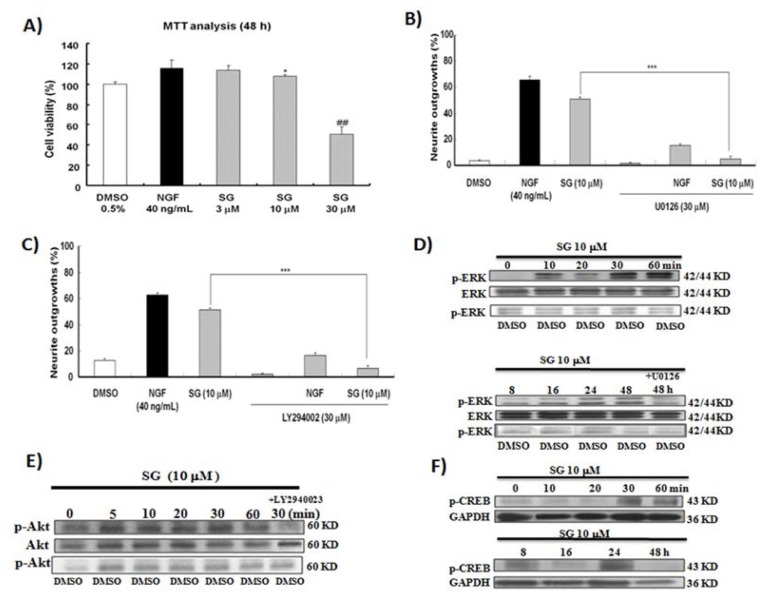
MTT analysis, effects of ERK and PI3K inhibitors on the neurite outgrowth of PC12 cells induced by SG and Western blot analysis results. (**A**) Results of MTT analysis; (**B**) The ERK inhibitor U0126 reduced the neurite outgrowth of PC12 cells induced by SG; (**C**) The PI3K inhibitor LY294002 inhibited the neurite outgrowth induced by SG; (**D**) Time-course of ERK phosphorylation after treating SG; (**E**) Time-course of Akt phosphorylation after treating SG; and (**F**) Time-course of CREB phosphorylation after treating SG. *, ^##^ and *** indicate significant differences relative to the control at *p* < 0.05, *p* < 0.01 and *p* < 0.001, respectively.

SG promoted cell generation at 3 and 10 μM but significantly reduced cell viability at 30 μM (Figure 5A). Hence, the best SG concentration for cell survival and NGF mimicking activity is 10 μM. The inhibitors of MEK and PI3K, which are U0126 and LY294002, respectively, significantly inhibited the neurite outgrowth induced by SG. Treatments with U0126 and LY294002 led to reductions in the neuritogenesis induced by SG from 52% and 53% to 8% and 6%, respectively (Figure 5B,C). Western blot analysis was performed to confirm these results at the protein level. In early time, SG could increase ERK1/2 phosphorylation. The phosphorylation of ERK started at 10 min and peaked at 30 min. In addition, the phosphorylation of ERK increased at 24 and 48 h again (Figure 5D). Furthermore, we investigated the phosphorylation of CREB induced by SG. It was significantly up regulated by SG at 30, 60 min and 8, 24 h (Figure 5F). Akt phosphorylation by SG began to increase at 5 min and peaked at 30 min (Figure 5E). These results indicate that PI3K/ERK/CREB signalling maybe involved in the SG-induced neuronal differentiation of PC12 cells.

NGF targeted TrkA and activated the RAS/RAF/MAPK downstream signalling cascades to produce neuritogenic activity. The lysophosphatidic acid essentially enhanced NGF-induced AMPK and Akt signals through the extracellular domain of TrkA. SG was different from them. It did not target TrkA but could activate PI3K/Akt/ERK/CREB signalling cascades to produce neuritogenic activity.

## 3. Experimental Section

### 3.1. Extraction and Isolation

The head of *I. elongate* was purchased in Hangzhou, Zhejiang Province, China. The sample (dry wt: 158.6 g) was powdered and extracted in MeOH (2 L) for 48 h at room temperature with stirring. The extraction was partitioned between EtOAc and H_2_O. The active EtOAc layer was concentrated to obtain 712.4 mg of the dried sample. The sample was chromatographed on silica gel (200–300 mesh, Yantai Chemical Industry Research Institute, Yantai, China) eluted with CHCl_3_/MeOH (100:0, 99:1, 95:5, 50:50) to yield 18 fractions. The active sample (4.8 mg) eluted with CHCl_3_/MeOH (95:5) was separated by HPLC (Develosil ODS-HG-5 (ϕ10/250 mm), Nomura chemical, flow rate: 3 mL/min, 80% to 100% MeOH/H_2_O in 60 min) to obtain MG (1.7 mg, *t*_R_ = 29.3 min).

MG: a colourless powder, ^1^H NMR (500 MHz, CDCl_3_): δ = 4.21 (dd, 1H, *J* = 4.3, 11.5 Hz), 4.14 (dd, 1H, *J* = 5.9, 11.5 Hz), 3.93 (m, 1H), 3.69 (dd, 1H, *J* = 4.3, 11.5 Hz), 3.60 (dd, 1H, *J* = 5.9, 11.5 Hz), 2.35 (t, 2H, *J* = 7.8 Hz), 1.62 (m, 2H), 1.26 (m, 20H), 0.88 (t, 3H, *J* = 7.0 Hz), ESI-TOF-MS *m*/*z* 325 [M + Na]^+^.

### 3.2. Chemical Analysis

NMR spectra were recorded on a Bruker AV III-500 spectrometer (Bruker, Billerica, MA, USA). The chemical shifts in δ (ppm) were referenced to the solvent peak of δ_H_ 7.26 for CDCl_3_. The chemical shifts given in δ (ppm) and signals were described as singlet (s), triplet (t), doublet of doublets (dd), multiplet (m). HR-ESI-TOF-MS was performed on an Agilent Technologies 6224A accurate mass TOF LC/MS system (Santa Clara, CA, USA). All of the solvents used were of analytical grade.

### 3.3. Synthesis Method

All of the glyceride derivatives (**1a**–**1i**) were prepared via 1,2-isopropylidene protection, esterification with alkyl acid and deprotection using glycerol as a starting material [24]. *N*-(2,3-dihydroxypropyl) stearamide (**2a**) was synthesised from 1,2-dihydroxy-3-aminopropane and stearic acid using EDC as an activating reagent [25]. The experimental details are presented in the supplementary information.

### 3.4. Bioassay of NGF Mimicking Activity in PC12 Cells

Biological activity was tested according to the methods described in our previous paper [26]. PC12 cells (20,000) were seeded in every well of a 24-well microplate and cultured under a humidified atmosphere of 5% CO_2_ at 37 °C. The medium was replaced by 1 mL of serum-free Dulbecco’s modified eagle medium (DMEM) containing a test sample or DMSO (0.5%) after 24 h. NGF was used as a positive control. Morphological changes in the cells were observed under a phase-contrast microscope. About 100 cells were observed from a randomly chosen area. A cell bearing neurite outgrowth longer than the diameter of cell body was identified as a positive cell. This process was repeated three times. Significant differences amongst groups were determined by ANOVA, followed by two-tailed multiple Student-Newman-Keuls *t*-tests using SPSS biostatistics software (IBM, Armonk, NY, USA). Values with *p* < 0.05 were considered significant. Independent experiments were repeated three times. Each value represents mean ± SEM of three replicates. ** or *** indicate significant differences relative to the control at *p* < 0.01 or *p* < 0.001, respectively.

### 3.5. MTT Assay

The cells were incubated with SG at concentrations of 3, 10 and 30 μM for 48 h. The medium was then replaced with 500 μL of serum-free DMEM containing 200 μg/mL MTT, and the plate was cultured in an incubator at 37 °C for 2 h. Finally, the medium was completely removed and 200 mL of DMSO was added to each well to solubilise the formazan crystals. The resultant formazan was detected at 570 nm using a plate reader (Bio-Tek instruments Inc., Winooski, VT, USA). All experiments were repeated three times.

### 3.6. Inhibitor Test

Cells in a 24-well microplate were pre-cultured with inhibitors for 30 min. Medium (500 μL) that contained the test sample or DMSO was then added to the wells. Morphological changes in the cells were observed after 24 and 48 h of treatment. Samples for Western blot analysis were collected at appropriate time points.

### 3.7. Western Blot Analysis

Western blot analysis was performed as described previously [18]. Briefly, 2 × 10^6^ cells were seeded in a 6 cm dish containing 5 mL of DMEM and incubated for 24 h. Serum-free DMEM containing SG at concentrations of 3, 10 and 30 μM was then added to the wells, and the dish was further incubated for appropriate times. To prepare a protein sample, the cells were gathered in lysis buffer and sonicated. The supernatant was removed as protein sample after centrifugation. About 15 μg of the protein sample was conducted by SDS-PAGE after protein concentration determination and then transferred onto a PVDF membrane. The membrane was incubated with primary and secondary antibodies. Antigen was visualised by chemiluminescent substrates (Beijing Cowin Biotech Company, Beijing, China). The primary antibodies used in this study included anti-phospho-p44/42 MAPK (Thr202/Tyr204), anti-p44/42 MAPK (ERK1/2), anti-phospho-CREB (Ser133), anti-Akt (Cell Signalling Technology, Boston, MA, USA), anti-Akt1 (phosphor S473) (Abcam, Hong Kong, China) and GAPDH (Beijing Cowin Biotech Company, Beijing, China) antibodies. The secondary antibody used was horseradish peroxidase-linked anti-rabbit IgG antibody (Beijing Cowin Biotech Company, Beijing, China).

## 4. Conclusions

In the present study, a bioassay system of PC12 cells was used for the screening, separation and purification of MG from the head of *I. elongate*. The structure of the compound was determined by comparison of its ^1^H NMR and MS data with those reported in the literature. Neurite outgrowth of PC12 cells was significantly induced by MG at 10 μM. To understand the SAR of MG, a series of monoglyceride derivatives was designed and synthesized. SG, which induced 57% of the neurite outgrowth of PC12 cells at 10 μM, was determined to be a lead compound. The mechanism of action of SG was investigated using PC12 cells via specific inhibitor experiments. Western blot analysis was then performed to confirm the results. MEK and PI3K inhibitors significantly decreased the neurite outgrowth induced by SG, and SG significantly increased phosphorylation of CREB. These results suggest that SG-induced neuritogenic activities depend on the activation of the PI3K/ERK/CREB signalling pathways in PC12 cells.

## Abbreviation

AD: Alzheimer’s disease
BBB: blood brain barrier
CREB: cAMP responsive element-binding protein
DMEM: dulbecco’s modified eagle medium
DMSO: dimethyl sulfoxide
ERK: extracellular regulated protein kinases
MEK: mitogen-activated kinase/ERK kinase
MG: 1-*O*-(myristoyl) glycerol
MTT: 3-(4,5-dimethylthiazol-2-yl)-2,5-diphenyltetrazolium bromide
NGF: nerve growth factor
NMDA: *N*-methyl-d-aspartic acid
PI3K: phosphatidylinositol-3 kinase
SAR: structure-activity relationship
SG: 1-*O*-(stearoyl) glycerol
